# Time Series Data Analysis of Wireless Sensor Network Measurements of Temperature

**DOI:** 10.3390/s17061221

**Published:** 2017-05-26

**Authors:** Siddhartha Bhandari, Neil Bergmann, Raja Jurdak, Branislav Kusy

**Affiliations:** 1 School of ITEE, University of Queensland, Brisbane 4072, Australia; siddhartha.raj.bhandari@gmail.com; 2 CSIRO/Data61, Pullenvale 4069, Australia; raja.jurdak@data61.csiro.au (R.J.); brano.kusy@data61.csiro.au (B.K.)

**Keywords:** wireless sensor networks, time series analysis, interpolation, forecasting, temperature, environmental monitoring

## Abstract

Wireless sensor networks have gained significant traction in environmental signal monitoring and analysis. The cost or lifetime of the system typically depends on the frequency at which environmental phenomena are monitored. If sampling rates are reduced, energy is saved. Using empirical datasets collected from environmental monitoring sensor networks, this work performs time series analyses of measured temperature time series. Unlike previous works which have concentrated on suppressing the transmission of some data samples by time-series analysis but still maintaining high sampling rates, this work investigates reducing the sampling rate (and sensor wake up rate) and looks at the effects on accuracy. Results show that the sampling period of the sensor can be increased up to one hour while still allowing intermediate and future states to be estimated with interpolation RMSE less than 0.2 °C and forecasting RMSE less than 1 °C.

## 1. Introduction

Wireless Sensor Networks (WSNs) allow dense spatiotemporal measurement of environmental phenomena such as temperature, humidity, solar radiation and rainfall [1] which in turn can be used to better understand local environmental conditions and processes. However, low-cost WSNs are also characterized by the resource constrained nature of the WSN hardware. Limited available energy for data sensing, storage and transmission is a common constraint in WSNs in remote areas where mains power in unavailable or uneconomical to access. Sensor nodes are typically battery powered, where node lifetime is determined by battery lifetime. Indefinite operation can be achieved with energy harvesting using technologies such as solar cells, but energy efficiency is still a key factor in determining the cost of deployment since more energy use means larger and more expensive rechargeable batteries and solar cells.

The spatial extent, spatial density and sensing frequency of the WSN nodes is partially determined by the scientific purpose of the deployment, but they will also be determined by the ability to model the processes which generate the environmental data in sufficient detail to be able to interpolate data values between sensed readings, both in time and space. If data can be accurately estimated between readings, then the frequency of making readings can be reduced, which in turn reduces the energy requirements and the deployment cost of the system, while increasing its lifetime. Previous work has not investigated the quantitative effects of reducing sampling frequency on the accuracy of both interpolated and predicted values. The optimal sampling interval will depend on the parameters being sensed, the environment in which they are sensed, the specific features of the sensors, and the scientific requirements for accuracy. This paper demonstrates the use of a data-driven method for determining sufficient sampling intervals through analysis of several specific sensor deployments. While we use temperature as a use case, many features of our approach are generalizable to other sensing modalities.

This paper first investigates the nature of temperature readings in a large scale WSN deployment in Springbrook, Australia [2]. Around 175 microclimate sensor nodes have been deployed for more than 5 years, and they have recorded temperature readings (as well as other environmental phenomena) every 5 min during this time. This provides a rich source of data for further analysis. For this paper, just one week of data has been explored, since there is a significant cost involved in data cleaning and checking prior to statistical analysis. The robustness of results would be improved if the analysis was applied to a larger portion of the data.

In this paper, the temporal dynamics of the temperature recorded by the WSN is analyzed in detail, with a view to answering two questions. Firstly, if the interval between sensing events is increased, how accurately can temperature be interpolated between the sensor readings. Longer sensing intervals will reduce the consumed energy, and hence reduce deployment cost or extend deployment lifetime. Secondly, if real-time readings of temperature are needed, for how long can future values of temperature be accurately extrapolated without needing instantaneous data transmission.

This paper addresses two research questions. Firstly, it analyzes the reduction in measurement accuracy if the sampling interval is extended with temperature interpolated between these values. Also different interpolation methods are compared. 

Secondly, we model the temperature phenomenon as a stochastic process and analyse it using a time series modelling framework [3], and use this analysis to determine how the short-term predictability of future temperature is affected by sampling interval, and extrapolation technique. 

The rest of the paper is organized as follows: Section 2 reviews the related literature. Section 3 explains the data used, Section 4 examines the first research question about the effect of sampling interval on temperature measurement accuracy, Section 5 repeats the analysis for a different data set, Section 6 explains time series modelling as background for the second research question, Section 7 answers this research question about future temperature prediction, and Section 8 concludes the paper.

## 2. Previous Work

WSN have the potential to revolutionize environmental sensing, providing high spatial and temporal resolution data [4]. Recent deployments include personal environment monitoring [5], city monitoring [6], building monitoring [7], ocean exploration [8] and toxic gas monitoring [9]. 

However, the nature of the measured phenomena are not always well understood. Environmental phenomena can vary at very small spatiotemporal scales [6,10]. Exhaustive spatiotemporal study of the behaviors of such dynamic phenomena requires deployment of an adequate number of sensor nodes and effective collection of data. 

In terms of temporal resolution, various ad hoc schemes have been proposed to optimize sampling frequency, e.g., in [11] soil moisture is sampled more frequently near rain events to give more useful data, however such techniques have not considered the detailed statistical nature of the signals.

Techniques have been proposed for spatially interpolating values within a sensor field [12,13,14,15] but these generally assume a smooth gradient across the sensor deployment area, and the techniques have not been well verified in real deployments. Most of the aforementioned references did not consider statistical behavior of the environmental phenomena or they assume process stationarity [15]. Liu et al. [16] also investigate spatially clustering nodes and reducing sampling interval by having only one sample report from a cluster each sample interval. The same effect could be achieved by simply reducing each cluster to a single node. Also, their spatial redundancy techniques have not been tested on real data, only on synthesized data.

Use of formal time series analysis in sensor networks has been reported by several researchers. Law et al. [17] use time-series modelling to decide the confidence levels for future samples, and skip the future readings if the values are likely to be accurate enough. However, this requires substantial processing (adjusting time series models continuously for each new sample), and it requires full rate sampling for some time after skipping samples. At best, it reduces the number of required samples by less than 50%.

In [18], Le Borgne et al. use time series prediction for future estimation of samples, so that some data transmission can be suppressed. They present a useful algorithm for selecting a suitable time series, but savings are only achieved for data transmission. The sensors still need to sample data at the full rate. Miranda et al. [19] use autoregressive models to predict samples based on spatially nearby sensors, however, their work does not investigate how to decide upon the optimum sample rate. Liu et al. [20] also present a method for suppressing the transmission of data samples if the receiver is able to accurately forecast samples based on time series models. Sensors are still required to sample data regularly. This method does not allow sampling intervals to be increased. Recently, Aderohunmu et al. [21] have also used similar time-series modelling for forecasting future sample values so that data transmission can be suppressed. Amidi [22] has used ARIMA modelling for the smoothing of noisy data and for interpolating missing data samples in a series, but again has not analysed the best sample rate to provide accurate data interpolation.

Pardo et al. [23] investigate a neural network model for predicting future temperature in an indoor environment for use with intelligent air-conditioning. Their neural network predictors perform considerably worse than Bayesian predictors (although the authors claim there is little practical difference), but their work does not investigate the effect of different sampling intervals.

Liu et al. [16] propose on-sensor temporal compression of data by only transmitting a dynamically computed subset of data (with linear interpolation between these). This reduces the quantity of transmitted samples, but not the sampling interval of the sensors, and also increases the latency before receiving measurements. 

Tulone and Madden [24] propose a system called Probabilistic Adaptable Query (PAQ) system which develops an AutoRegressive (AR) time series model for every node for predicting future values. If the future predictions based on past transmitted values are below some threshold, then no new data is transmitted. Once this threshold is exceeded, new data is transmitted. Data still needs to be sampled at high temporal resolution, and there is no investigation of what the best sampling interval should be. They also propose round-robin scheduling on sensors in spatial clusters.

In general these previous works have used time series analysis to model the statistical behavior of the data. They have been used for outlier and anomaly detection, and for separating the underlying trends from noisy signals. They have been used for suppressing data transmissions when forecast values are close to the measured values. However, with such systems, there has been no reduction in the sampling interval, just in the transmitted data. Energy use consists of three main components. Firstly every time data needs to be sampled, the sensor node needs to wake up, wait for the sensor node and sensing transducer to stabilize, undertake any computational tasks (such as calibrating readings, or comparing against predicted estimates of values), and possibly transmitting data to the data sink. Previous work still requires the sensor to wake up, stabilize and compute at high sampling frequency. Even if the energy to wake up, stabilize and compute is relatively small compared to transmission costs, as would be the case for a temperature sensor, reducing the sensing frequency, and hence the number of wake up times, will have a direct impact on sensor lifetime. Substantially more energy can be saved in the sensor sampling interval can be extended without compromising the scientific usefulness of the collected data. Previous work has not used time series analysis to analyse the accuracy of both interpolated and extrapolated data values as the sampling period is varied. This analysis can help a sensor network designer to set a sampling rate that satisfies the required error limit whilst reducing energy consumption.

In this work, no behavioral assumptions of the process are made and all analyses are validated with proper statistical tests. This analysis will allow insights into the required sampling intervals for long-term deployments with moderate accuracy requirements.

It is worth noting that several papers, e.g., [16,24], reduce sampling intervals by round robin scheduling of nodes with a spatial cluster of highly correlated nodes. In this paper, only sampling within a single time series is investigated, although we expect to address spatial redundancy in our future work.

## 3. Temperature Data from Springbrook WSN Deployment

This section describes one set of temperature data that used for this study, and presents some simple empirical observations. Situated in southeast Queensland, the Springbrook WSN deployment consists of 175 sensor nodes monitoring temperature, pressure, humidity, wind, and several other environmental parameters with a sampling period of 5 min, and it has been operating since 2008 [2].

**Figure 1:**
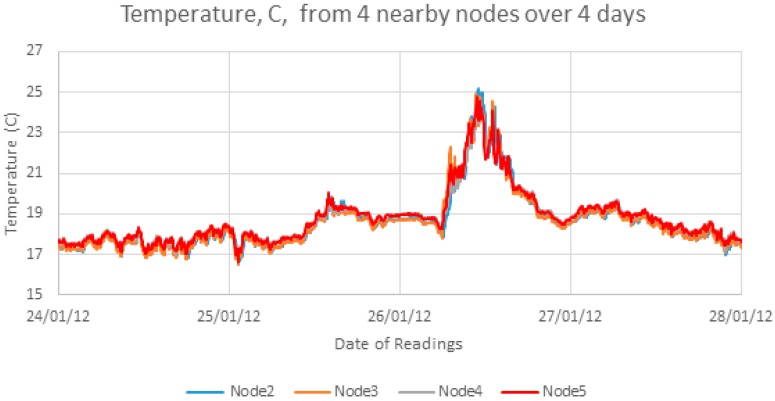
4 day time series plot of four nearby sensors.

Figure 1 shows four days of data from four sensors in the deployment which shows that the temperature patterns are highly correlated between nearby sensors. This means that interpolation and prediction results from one sensor node should be representative of results from all nodes in that deployment. However, the temporal pattern over the week does not always show a clear daily pattern.

**Figure 2:**
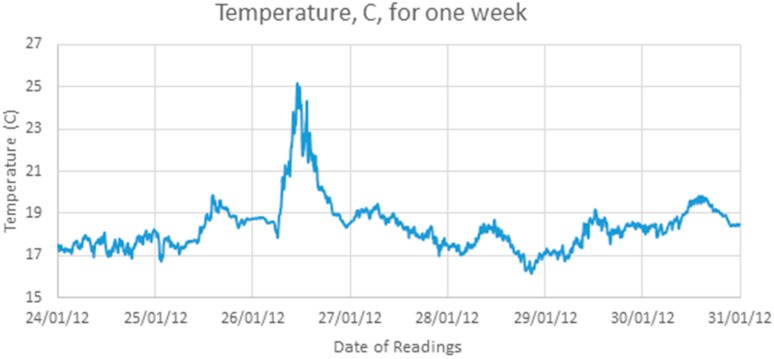
One week of samples from one sensor.

Figure 2 shows the readings of one sensor over one week which shows that the temperature does not rise and fall smoothly over the course of a day but has a significant component of noise.

**Figure 3:**
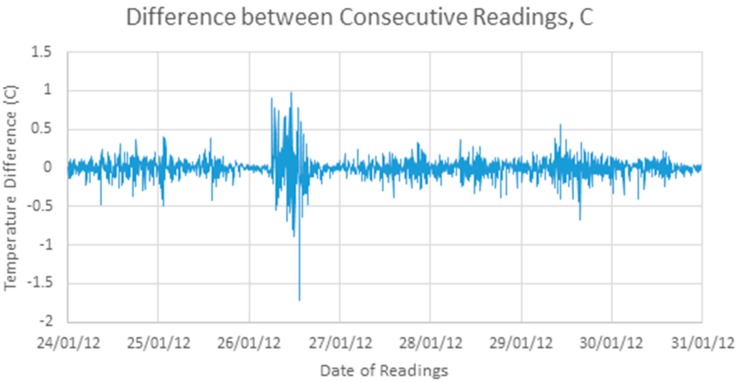
One week of difference values.

Figure 3 shows a differenced version of the signal, as given by Equation (1):

*Y’*(*t*) = *Y*(*t*) − *Y*(*t* − 1) (1)

On first observation, this differenced signal does not have any clear structure, but appears largely random. Simple statistical analysis shows a mean close to zero and a standard deviation of 0.14 °C. 

## 4. Accuracy versus Sampling Interval

As mentioned earlier, energy can be saved and sensor lifetime extended if the interval between sensor readings is extended. In this first experiment, the sensing interval is extended from the existing 5 min intervals to intervals of 10 min, 15 min, 20 min, 30 min, 45 min, 60 min, 90 min and 120 min by selecting appropriately spaced samples from the 5-min data for one sensor over one week. Values at the intervening 5 min intervals are then interpolated, and the RMSE (root-mean-square error) and MAE (mean absolute absolute error) of the interpolated values are calculated. Two different interpolation algorithms are chosen. The first method uses linear interpolation between the sampled points, and the second method uses a cubic spline between the sample points. Table 1 shows the RMSE and MAE of interpolated values, and the 99th percentile absolute error when the various interpolation methods are applied to the one week sequence shown in Figure 2. 

**Figure 4:**
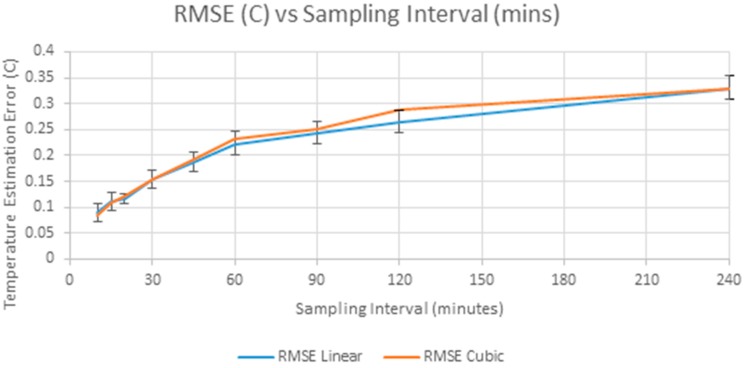
RMSE of linear and cubic interpolation showing 95% confidence interval of RMSE Linear.

Figure 4 shows the growth of error with increasing sample intervals. The 95% confidence interval for the RMSE of linear interpolation is also shown in Figure 4, and the difference between linear and cubic interpolation is not significant within these confidence intervals. Except at smaller sampling intervals, cubic spline interpolation gives poorer results, and so linear interpolation is preferred.

These results show that with linear interpolation, the MAE remains below the standard deviation of the difference signal (0.14 °C) in Figure 3 when the sampling interval is extended to 60 min. Alternatively, if the accuracy requirement was that 99% of interpolation errors have an absolute magnitude of less than 0.5 °C then the sampling interval can be extended to 20 min.

It should be stressed that these results apply to this particular deployment. The general result, however, is that statistical analysis of sampled data over an initial deployment at relatively high sampling rate can give insights into a lower long-term sampling rate which does not significantly sacrifice accuracy.

## 5. Repeating for Another Data Series

**Figure 5:**
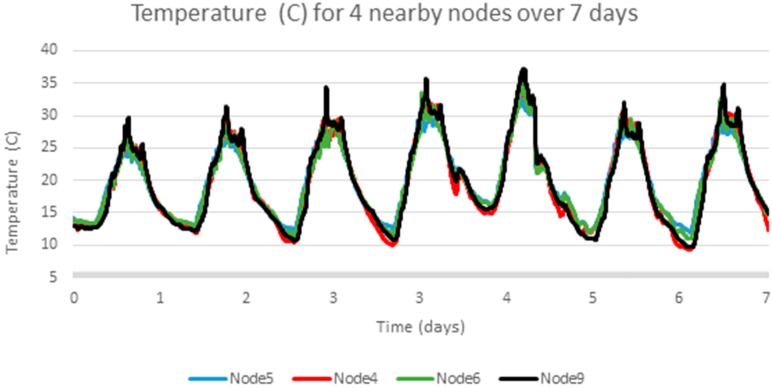
Adjacent sensor readings for a second experiment.

**Figure 6:**
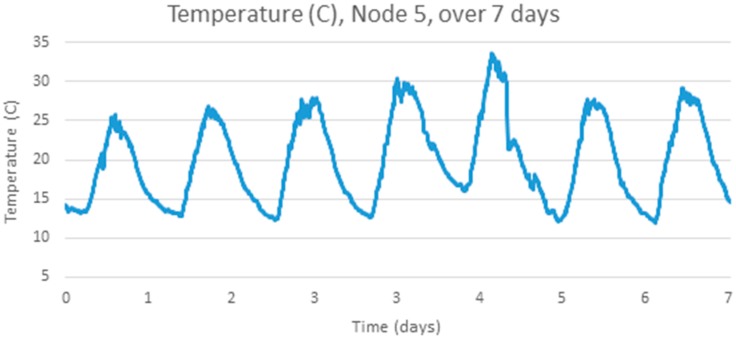
Detailed Readings for Node 5.

**Figure 7:**
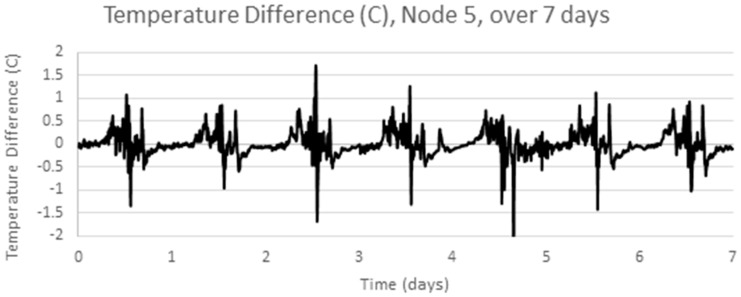
Temperature Difference, Node 5 over 7 days.

The analysis above is repeated for another temperature data set using a different set of sensor hardware, a different physical location (a mine rehabilitation and revegetation site) and a different time of year (December 2013), again with samples every 5 min [25]. Figure 5 below shows four adjacent sensors over a one week period, Figure 6 shows one signal, Node 5, in detail, which has a clear cyclic pattern. Figure 7 shows the differences between consecutive signals over 7 days. The signal appears mostly like a random noise signal, centred on zero. The variance of the noise is not constant, but also varies cyclically with higher variances in the middle of the day. The standard deviation of the temperature difference is around 0.3 °C.

Table 2 repeats the analysis of how well linear interpolation and cubic spline interpolation can estimate intermediate temperatures if the sampling interval is reduced to 10 min, 15 min, 20 min, 30 min, 60 min, 690 min, 120 min or 240 min.

Again linear interpolation gives better estimates at smaller sampling intervals up to 60 min. For sampling intervals over 60 min, there is a small advantage for cubic spline interpolation. The results also show that the sampling interval can be extended to about 60 min without the errors in the interpolated values exceeding 0.3 °C, which is the standard deviation of the difference signal between consecutive samples.

## 6. Time Series Analysis of Random Processes

The next experiment involves forecasting future values of temperature based on past samples. Liu et al. [20] described a system for saving sensor transmission energy when real-time estimates of temperature are needed. Samples are taken at regular intervals, and at each interval both the sender and the receiver calculate an estimated value based on the past time series. If the actual sensed value at the transmitter is within an error margin (say 0.5 °C) then no data is sent, and the receiver uses the forecast estimate. Once the error exceeds the error limit, then the actual current value plus any recent past values needed for future forecasting are sent. Liu et al. show a reduction in transmitted data of 70% with a corresponding reduction in energy use. However, their work uses indoor temperature readings with a very smooth behavior. We are interested if such a forecasting approach also works in a much more variable outdoor environment. Forecasting of future values uses an ARIMA process model and the subsequent sections explain the theoretical background behind such forecasting before such techniques are applied to our data.

### 6.1. Time Series and Stochastic Process

Due to the lack of complete knowledge of the complex underlying physical processes that generate local climate, environmental phenomena are in general modelled as stochastic processes [26]. A stochastic process varying in time is characterized by the sequence of a random variable. Any time sequenced realization of such a process is called a time series. Time series analysis involves a range of investigations of the behavior of the observed stochastic process. Such analyses reveal structural behavior of the process that can be used to fit a suitable statistical model and understand short-term and long-term behaviour. Time series analysis is widely employed in areas such as signal processing, business processes and economic modelling, and there are many references which explain the concepts in detail [27,28,29].

Typically, in time series analysis, a process *Y*(*t*) is assumed to consist of several sub components: a trend, *µ*(*t*), a periodicity *P*(*t*), seasonality, *P*(*t*), and a random shock *e*(*t*), as shown in Equation (1). The trend component represents a deterministic tendency such as long term global warming; a periodicity represents regularly repeating behavior such as diurnal temperature variations; seasonality represents longer term patterns such as summer and winter, and the random shock captures the effects of local short term changes which are not explained by the longer term patterns:

(2) Y(t)=μ(t)+P(t)+S(t)+e(t)

If the properties of a process vary with time, then it is difficult to predict future values from its observed time series *Y*(*t*) and such a process is called a non-stationary process. Most environmental phenomena fall in this category. In order to analyze a random process and perform state estimation, some sort of stationarity assumption needs to be made. In general, a second order stationarity assumption is made which assumes that the mean and the variance characteristics of the process do not change over time. 

### 6.2. Time Series Model Development Strategy

**Figure 8:**
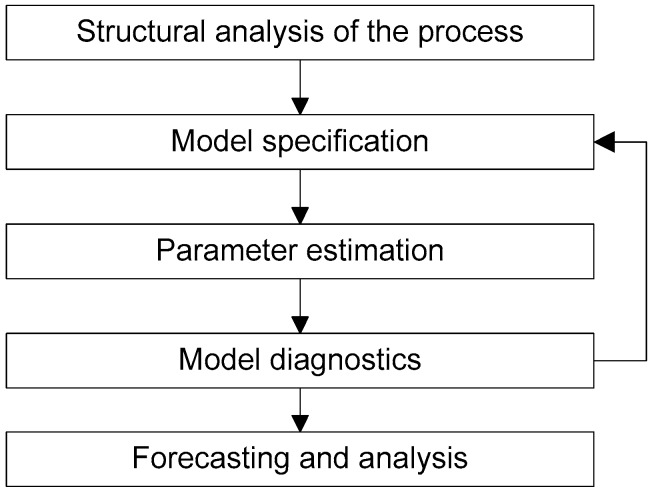
Time series model development strategy.

Time series model development involves estimating a process characterizing components mentioned in Equation (2) with several sequential steps as shown in Figure 8. This generic time-series analysis framework is also known as Box-Jenkins time series modelling [28]. Structural analyses study the sample autocorrelation function and examine the stationarity property of the process. 

#### 6.2.1. Model Specification

In general, the current state of any random process may depend on time, its past states, and some random shocks or a combination of these. Such dependencies of the observed series need to be extracted. Linear or nonlinear regression captures the trend component of the process. Dependencies with previous states can be captured by regression of the current state with the previous state and the effect of random shocks can be captured by involving noise components. 

There are many different possible time series modelling approaches, but the most general of these is the Auto Regressive, Integrated, Moving Average (ARIMA) model. Stationarity of time series can be determined from the analysis of sample autocorrelation function and conducting an Augmented Dickey-Fuller (ADF) unit root test [28]. If the time series is found to be non-stationary, transformation of the series can be performed that makes the series stationary. Logarithmic and power transformation and series differencing are the most commonly used transformation approaches. If the difference is taken to make the time series stationary, then the model is an Integrated model (i.e., ARIMA rather than ARMA). The order of the differencing is represented by a parameter d.

The ARIMA model specification involves finding suitable autoregressive (AR) and moving average (MA) sub components of the Integrated model. The model represented in Equation (2) and can then be specified as in Equation (3):

(3) $$Y_{t}=\mu +\phi_{1}Y_{t-1}+\cdots +\phi_{p}Y_{t-p}{+e}_{t}-\theta_{1}e_{t-1}-\cdots -\theta_{q}e_{t-q}$$

Parameters specify deterministic (*µ*), autoregressive (*φ*), moving average (*θ*), and error (*e*) components. *p* and *q* represents the orders of AR and MA components which are determined by analyzing sample autocorrelation, and extended autocorrelation function of the time series. Overall, the time series is then modelled by an ARIMA (*p*, *d*, *q*) model.

#### 6.2.2. Parameter Estimation

After specifying differencing to achieve stationarity and specifying the AR and MA orders, the next step is the estimation of the parameters involved in Equation (3). For most random processes, parameters *φ_i_* and *θ_i_* are estimated using a Least Square (LSE) or Maximum Likelihood (ML) estimator. These parameters can then be used to estimate future values of the series 

#### 6.2.3. Model Diagnostics

Model specification deals with examining the goodness of the fit of the model parameters. Analysis of the residuals and over-parameterized models are two approaches used for validation. If residuals obtained after fitting a model fit a Gaussian noise distribution, then the model is considered to be valid. Over parameterizing models involve internationally over fitting model with higher orders of *p* and *q*. If the over fitted model doesn’t show significant improvement in its residuals, the fitted model is considered to be valid. 

#### 6.2.4. Time Series Forecasting

After fitting a suitable model, the future state of the time series can be forecast. These future values can themselves be used to estimate further future values of the series. The forecasting power of the time series model is based on how many future sample values can be estimated with some desired accuracy.

## 7. Forecasting Experiments

As mentioned earlier, forecasting of future values can reduce the transmission energy for real-time temperature modelling. Analysis of the mine site temperature data (from Figure 6 above) is undertaken to estimate the forecasting accuracy of future samples.

The time series analysis in Section 6 uses standard methods to characterize the physical process. This section proposes a mechanism that uses the results of the time series analysis to identify the best sampling interval for a sensor deployment. We also observe what level of prediction improvement is gained by use of ARIMA models.

Environmental time series are usually non-stationary and require data cleaning to deal with missing data due to energy failures or other causes. The non-stationary nature is addressed by applying differencing and checking that the difference signal is stationary, as described in Section 7.1. The data used here has been manually checked the series here have been cleaned of any missing or repeated data (which was less than 1% of the data samples).

### 7.1. Structural Analysis of Time Series

Data analyses in this paper are primarily done in R [30], specifically using the package developed in [31]. Microsoft Excel and MATLAB are used for some data formatting and data plotting.

**Figure 9:**
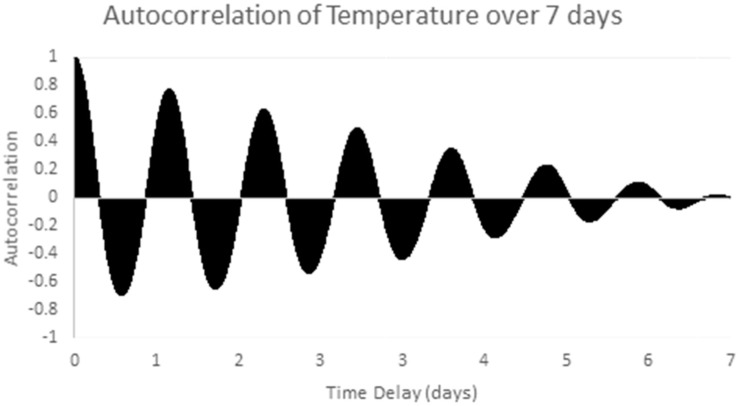
Sample autocorrelation of temperature in experimental data (5 min samples over 1 week).

**Figure 10:**
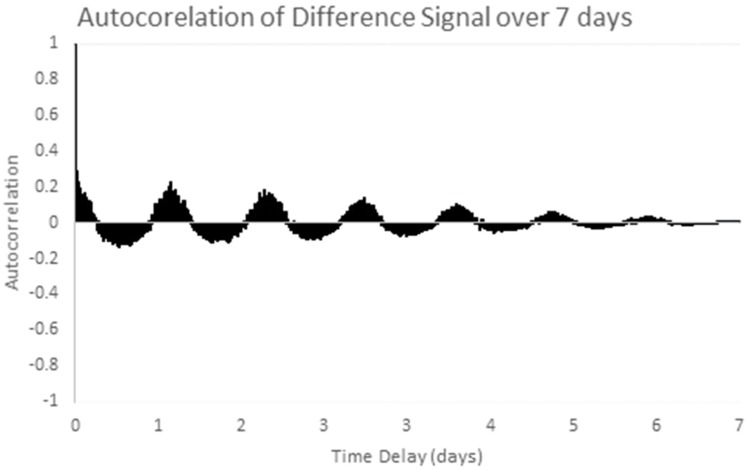
Autocorrelation of the differenced sample series over 7 days.

The chosen data series is the one week sample series shown in Figure 6 above. Stationarity is checked by examining the one week sample autocorrelation plot of the selected series, as shown in Figure 9. This autocorrelation plot has a clear structure which varies with the autocorrelation lag. Temperature patterns in one day are clearly correlated with the pattern the next day. This shows the clear presence of non-stationary (periodic) behavior in the series. After applying differencing, the time series in Figure 7 above was obtained. Figure 10 shows the autocorrelation of the differenced signal. Compared to the sample autocorrelation of Figure 9, the differenced series has an autocorrelation function which still has some regular structure, but the magnitude of the autocorrelation is less than 0.2 for all lags.

**Figure 11:**
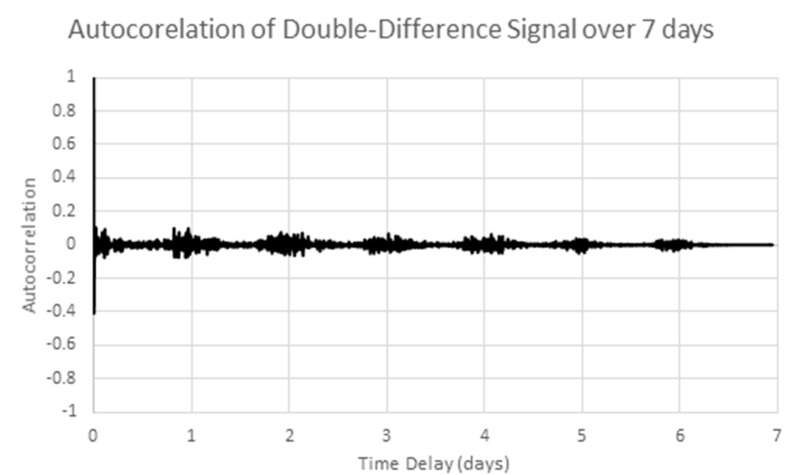
Autocorrelation of the doubly differenced sample series.

After applying one more round of differencing (a doubly differenced series) the autocorrelation in Figure 11 results which shows the double differences are uncorrelated. However, since single differencing gives the low autocorrelation values in Figure 10, the singly differenced signals will be used for further analysis.

### 7.2. Model Order Selection

As the series becomes stationary after differencing, an ARIMA model will be used for the time series model. As the average of the differenced series varies about zero, the expected value of the deterministic trend can be considered to be zero. The next step is to determine the orders of AR and MA components for the most suitable model. The Akaike Information Critera (AIC) are widely used criteria which trade off the increased accuracy of higher order models with the parsimonious use of fewer model components [21]. Using the “auto.arima” routine from the *forecast* package in R which tests many different models, AR and MA orders of the series are estimated for different sampling rates. Estimation of AR and MA orders for different sampling rates help us to examine how time series model varies with different sampling rate of the deployed sensors. Table 3 shows the models with the best AIC score based on the first three days of data as shown in Figure 8 above, for different sampling rates (i.e., for subsampled subsets of the original data). These different sampling rates capture different realizations of the process and specify different orders for the ARIMA models, however there is not any clear interpretation of how ARIMA model order varies with sampling rate, other than the fact that for this data set, 60 min sampling gives the simplest model. 

Experiments on other data (such as the data shown in Figure 2, or on different subsets of the week in Figure 8) show that the best ARIMA model order is not very consistent between different deployments or different periods and would need to be revised regularly when used for prediction. Rechecking and updating the best predictive model order once a week for each different sensor (rather than using a single model order for all deployments) would allow seasonal changes in model order to be tracked.

### 7.3. Forecasting

To test the forecasting ability of the time series models, the ARIMA models are used to forecast the remaining four days of data shown in Figure 6. In particular, the following procedure is used. For each sampling rate, the ARIMA model of the order shown in Table 3 is trained on three days of data, and then used to predict up to two hours forward from that point, e.g., for 5 min sampling, 24 future points are estimated, for 30 min four future points are estimated, and for 120 min, one future point is estimated. Then the 3 day training window is moved forward by 2 h, the models retrained, and the process repeated for the remainder of the four “testing” days of the sample. For sampling rates greater than 5 min, the future predictions at 5 min intervals are linearly interpolated between the future prediction points. For example, for 30 min sampling, the future prediction at 5 min is linearly interpolated between the last data point and the first predicted point.

Additionally, two other prediction models are used based on the 5 min sampled data. The “zero difference” model uses the last data point in the undifferenced series as the predictor for the next two hours. This is the same as using the mean (zero) of the differenced series as the predictor of the next difference. The “same difference” model linearly extrapolates from the last two data points in the undifferenced series, which is the same as assuming that the next difference value is the same as the current difference value.

**Figure 12:**
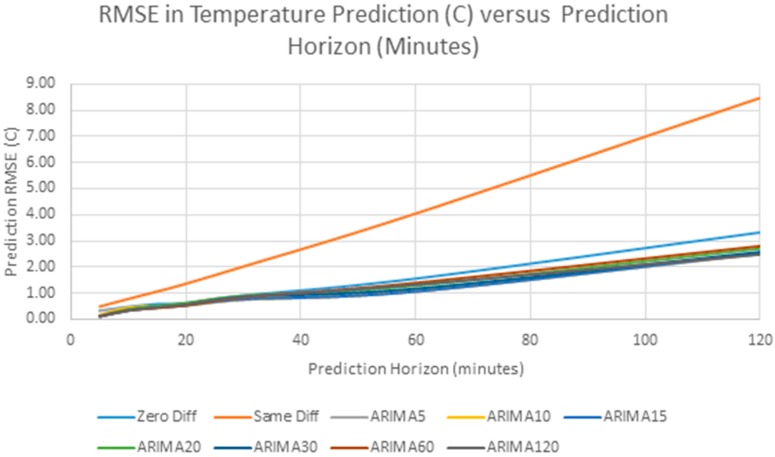
RMSE versus Prediction Horizon for Different Predictors.

The accuracy of the future predictions are measured by the RMSE of the predictions across the four days, and also the MAE of the predictions. Table 4 shows the results for RMSE and Table 5 shows the results for MAE. Figure 12 shows a plot of the RMSE for the different predictors versus the forecast time, where, for example “ARIMA5” means the ARIMA model with 5 min sampling interval.

**Figure 13:**
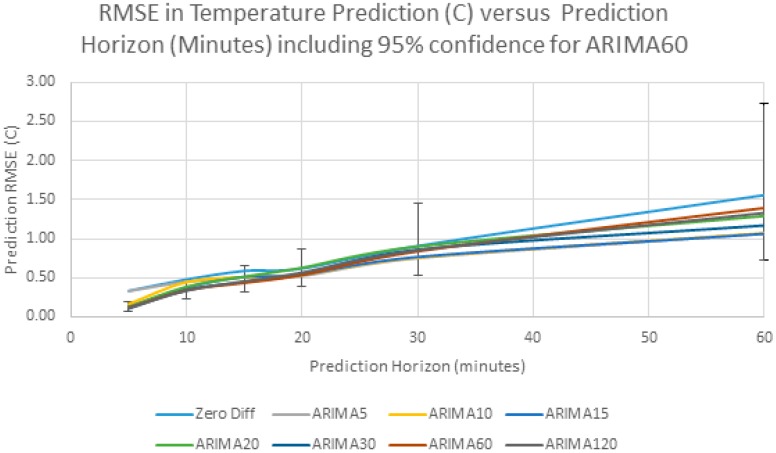
Detail of RMSE versus Prediction Horizon for Different Predictors with 95% confidence interval for ARIMA60.

Because the different predictors are difficult to distinguish in Figure 12, Figure 13 shows an expanded close up of the prediction up to 60 min, with the poorly performing linear extrapolation (Same Difference) excluded. Figure 13 also shows the 95% confidence interval for the ARIMA60 results, showing that the differences between predictors is small compared to the confidence interval.

As can be seen from this data, the RMSE in forecasting increases as we forecast further in the future and it exceeds 1 °C after about 60 min. This behavior can be explained by the sample autocorrelation function in Figure 8. The correlation between samples decreases steadily as the lag increases, and so, as predicted, the prediction error steadily increases. Another interesting observation from Figure 13 is that the forecasting error does not change significantly with sampling interval. The “Same Difference” or linear extrapolation method performs very poorly, and the “Zero Difference” method also performs worse than any of the ARIMA models. In this particular example, the ARIMA model prediction with 30 min sampling has lowest error. The differences between the ARIMA models with different sampling intervals is small, and it is expected that the differences are artifacts of the particular data series. However, a clear message is that prediction accuracy does not depend on high frequency data sampling.

## 8. Conclusions

In this paper, univariate time series analysis is performed on an environmental sensors array deployed for monitoring outdoor environmental temperatures. Statistical properties of the phenomenon are observed and a suitable time series model is fitted. After parameter estimation, evaluation of the forecasting error of the future temperature is performed with varying sampling period of the sensor. Interpolation between subsampled series is also performed, and linear interpolation is preferred to more complex cubic spline interpolation. Temperature can be interpolated with an RMSE accuracy of less than 0.2 °C while extending the sampling interval to 60 min. For prediction, an RMSE in prediction of less than 1 °C is possible if the sampling interval is extended to around 60 min.

Altogether, this detailed analysis shows than frequent temperature sampling (every 5 min) provides limited additional information over sampling at intervals up to 60 min. Such a down sampling can be helpful in extending the energy-limited lifetime of the sensor, and reducing the data storage requirements. 

This analysis has shown that it is not possible to state the best sampling interval for all deployments based on experiments from one deployment. Instead, determination of the best sampling intervals would need to be done on a case-by-case basis after some initial high-frequency sampling. Then detailed data analysis using the methods described above can be used to determine a suitable sampling interval for that particular deployment. Our planned future work will move from the required temporal resolution to look at the required spatial resolution for measuring sensor data across a geographical area.

## Figures and Tables

**Table 1:** Interpolation Error for Different Sampling Intervals (in °C).

Sampling Interval (Mins)	RMSE Linear	MAE Linear	RMSE Cubic	MAE Cubic	99% Linear	99% Cubic
10	0.0884	0.0528	0.0852	0.0519	0.3250	0.2893
15	0.1097	0.0664	0.1088	0.0669	0.4000	0.4037
20	0.1166	0.0755	0.1228	0.0793	0.4200	0.4496
30	0.1527	0.0937	0.1531	0.0962	0.5800	0.5709
45	0.1865	0.1152	0.1921	0.1190	0.6867	0.7410
60	0.2224	0.1335	0.2330	0.1430	0.8425	0.8753
90	0.2439	0.1566	0.2507	0.1629	0.9133	0.8774
120	0.2646	0.1720	0.2893	0.1882	0.9425	1.0206
240	0.3297	0.2161	0.3290	0.2215	1.2758	1.2189

**Table 2:** Interpolation Error (°C) for Different Sampling Intervals for Mine Data.

Sampling Interval (Mins)	RMSE Linear	MAE Linear	RMSE Cubic	MAE Cubic	99% Linear	99% Cubic
10	0.1746	0.0941	0.1751	0.0960	0.6740	0.6366
15	0.2085	0.1164	0.2185	0.1211	0.7554	0.8286
20	0.2342	0.1360	0.2487	0.1459	0.8862	0.9436
30	0.2723	0.1588	0.2846	0.1693	1.0099	1.0027
45	0.3664	0.2029	0.3694	0.2087	1.2578	1.3131
60	0.4655	0.2498	0.4635	0.2493	1.5781	1.5309
90	0.5837	0.3093	0.5762	0.3033	1.9658	1.8047
120	0.6057	0.3836	0.5840	0.3663	2.1344	2.0859
240	0.9780	0.6687	0.8121	0.5515	3.0073	2.7782

**Table 3:** AR and MA orders for different sampling rates.

Sampling Rate (Minutes)	Fitted Models
5	ARIMA(3,1,1)
10	ARIMA(2,1,2)
15	ARIMA(1,1,3)
20	ARIMA(1,1,3)
30	ARIMA(2,1,1)
60	ARIMA(1,1,0)
120	ARIMA(3,1,1)

**Table 4:** RMSE of Future Temperature Predictions in °C.

Forecast	Simple Models		ARIMA Models Sampling Intervals (Minutes)						
Time (Mins)	Zero Diff	Same Diff	5	10	15	20	30	60	120
5	0.33	0.49	0.33	0.17	0.13	0.13	0.11	0.12	0.12
10	0.48	0.78	0.45	0.45	0.38	0.39	0.35	0.34	0.34
15	0.59	1.07	0.51	0.51	0.51	0.51	0.45	0.44	0.46
20	0.62	1.36	0.53	0.54	0.54	0.63	0.54	0.54	0.57
30	0.91	2.02	0.75	0.76	0.77	0.90	0.86	0.84	0.85
60	1.56	4.05	1.07	1.07	1.06	1.29	1.17	1.39	1.33
120	3.32	8.47	2.50	2.52	2.52	2.71	2.58	2.80	2.48

**Table 5:** MAE of Future Temperature Predictions in °C.

Forecast	Simple Models		ARIMA Models Sampling Intervals (Minutes)						
Time (Mins)	Zero Diff	Same Diff	5	10	15	20	30	60	120
5	0.24	0.27	0.21	0.11	0.08	0.08	0.07	0.08	0.09
10	0.35	0.49	0.32	0.32	0.26	0.26	0.23	0.21	0.22
15	0.45	0.65	0.38	0.38	0.38	0.36	0.31	0.29	0.31
20	0.52	0.87	0.42	0.42	0.42	0.46	0.40	0.37	0.43
30	0.73	1.31	0.58	0.58	0.59	0.63	0.63	0.55	0.62
60	1.27	2.60	0.82	0.82	0.81	0.85	0.82	0.90	0.96
120	2.71	5.71	1.91	1.94	1.98	2.03	1.97	2.03	1.74
